# Prion Strains Differ in Susceptibility to Photodynamic Oxidation

**DOI:** 10.3390/molecules27030611

**Published:** 2022-01-18

**Authors:** Marie Kostelanska, Karel Holada

**Affiliations:** Institute of Immunology and Microbiology, First Faculty of Medicine, Charles University, 128 00 Prague, Czech Republic; marie.kostelanska@lf1.cuni.cz

**Keywords:** prion, PrP, TSE, strain, protein folding, phthalocyanine, photodynamic, PDI, singlet oxygen, prion inactivation

## Abstract

Prion disorders, or transmissible spongiform encephalophaties (TSE), are fatal neurodegenerative diseases affecting mammals. Prion-infectious particles comprise of misfolded pathological prion proteins (PrP^TSE^). Different TSEs are associated with distinct PrP^TSE^ folds called prion strains. The high resistance of prions to conventional sterilization increases the risk of prion transmission in medical, veterinary and food industry practices. Recently, we have demonstrated the ability of disulfonated hydroxyaluminum phthalocyanine to photodynamically inactivate mouse RML prions by generated singlet oxygen. Herein, we studied the efficiency of three phthalocyanine derivatives in photodynamic treatment of seven mouse adapted prion strains originating from sheep, human, and cow species. We report the different susceptibilities of the strains to photodynamic oxidative elimination of PrP^TSE^ epitopes: RML, A139, Fu-1 > mBSE, mvCJD > ME7, 22L. The efficiency of the phthalocyanine derivatives in the epitope elimination also differed (AlPcOH(SO_3_)_2_ > ZnPc(SO_3_)_1-3_ > SiPc(OH)_2_(SO_3_)_1-3_) and was not correlated to the yields of generated singlet oxygen. Our data suggest that the structural properties of both the phthalocyanine and the PrP^TSE^ strain may affect the effectiveness of the photodynamic prion inactivation. Our finding provides a new option for the discrimination of prion strains and highlights the necessity of utilizing range of prion strains when validating the photodynamic prion decontamination procedures.

## 1. Introduction

Prion disorders, also known as transmissible spongiform encephalopathies (TSE), represent a group of fatal neurodegenerative diseases affecting man and several other mammalian species. Within the same species, TSE can differ in the length of preclinical phase, clinical signs, speed of progression, location of brain lesions and also in biochemical properties of accumulated pathological PrP^TSE^ [1]. Most of the patterns seem to be explained by the existence of so-called prion strains represented by distinct self-propagating PrP^TSE^ conformations [2]. Prions are exceptionally resistant to conventional heat and chemical-based sterilization procedures [3]. The World Health Organization recommends the immersion of contaminated instruments in 1 M NaOH or 2% NaOCl for 1 h, followed by autoclaving at 134 °C for 1 h and subsequent routine sterilization. However, such harsh treatment is not appropriate for various medical instruments, and several less-damaging procedures have been studied, some with encouraging results [4,5]. Interestingly, prion strains notably differ in their resistance to inactivation. For example, the hamster Sc237 strain was shown to be five orders of magnitude more sensitive to acidic SDS treatment than human CJD prions [6]. Notable variation was also found in the thermostability of mouse-adapted prion strains [7]. Out of the two most commonly used laboratory mouse scrapie strains, lymphotropic RML and neurotropic ME7, the ME7 strain displays notably higher stability of PrP^TSE^ core to denaturation [8]. We have previously demonstrated inactivation of mouse RML prions by photodynamic treatment with disulfonated hydroxyaluminum phthalocyanine (AlPcOH(SO_3_)_2_) [9,10]. Whether the susceptibility of prions to photodynamic treatment varies among the prion strains is at present unknown. In this study, we utilized seven mouse-adapted prion strains (Fu-1, mvCJD, mBSE, ME7, 22L, 139A and RML). Two strains were of human origin, Fukuoka-1 (Fu-1) and mvCJD were derived from the tissues of Gerstmann-Sträussler-Scheinker syndrome and variant Creutzfeldt–Jakob disease patients, respectively [11]. Mouse-adapted BSE (mBSE) was derived from the tissue of cattle affected by bovine spongiform encephalopathy. The ME7, 139A, RML and 22L strains originated from sheep scrapie. The strains RML, 139A and 22L were isolates from the same sheep, but they followed varying passaging through goats (RML, 139A) or sheep (22L) before adapting to mice. The ME7 strain was derived from sheep independently [12]. The RML and 139A strains are believed to originate from the same mouse isolate, but were adapted to mice in separate laboratories and behave differently [13].

Within the prion field, the phthalocyanine (Pc) derivatives were first studied as potent anti-prion compounds able to cure cell cultures from prion infection and even prolong the survival of prion infected mice [14,15]. The anti-prion activity of Pc derivatives correlated with their self-association tendencies [16]. The mechanistic explanation of their effect predicted π–π interactions of planar Pc molecule with aromatic amino acid residues of PrP [16,17]. The direct binding of up to 10 Pc molecules to 1 molecule of recombinant hamster PrP with micromolar affinity was demonstrated by Dee et al. [18]. Pcs have been used in various photo-applications, including phototherapy of cancer, bioimaging and the photodynamic inactivation (PDI) of microorganisms [19,20]. Our laboratory was the first to utilize Pc for the PDI of prions [9]. The treatment of RML brain homogenate with AlPcOH(SO_3_)_2_ led to the disappearance of the PrP^TSE^ signal detected by Western blot, decreased its infectivity in cell culture assay and, importantly, also significantly decreased its infectivity in mouse bioassay [9,10]. The mechanism behind the photodynamic elimination of PrP^TSE^ epitopes and prion inactivation relies on phthalocyanine′s generation of singlet oxygen O_2_(^1^∆_g_) [10]. The reaction of O_2_(^1^∆_g_) with proteins is known to preferentially modify the side residues of five amino acids: W, Y, H, M and C [21].

The goal of this study was to evaluate the potential of three sulfonated Pc derivatives ZnPc(SO_3_)_1-3_, AlPcOH(SO_3_)_2_ and SiPc(OH)_2_(SO_3_)_1-3_ for the PDI of prions and to examine if the susceptibility of prions toward photodynamic treatment is prion strain dependent. We found significant differences both in the efficiency of Pc derivatives and in the susceptibility of the strains.

## 2. Results

### 2.1. Phthalocyanine Derivatives Characterization

#### 2.1.1. Differences in the Phthalocyanine Structure

**Figure 1:**
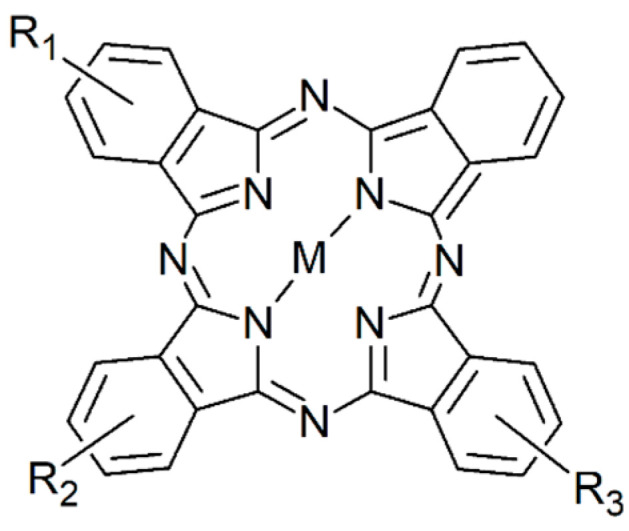
Structure of sulfonated metal phthalocyanines, where M represents coordinated central metal, and R_1_ = R_2_ = R_3_ indicates the position of SO_3_ substituents of peripheral benzene rings.

The Pc derivatives differed in the nature of the central metal atom (Al, Zn, Si), the level of the metal hydroxylation (from zero to two) and the number of substituting sulfonate groups on their peripheral benzene rings (Figure 1, Table 1). 

#### 2.1.2. Spectroscopic Properties and Production of Singlet Oxygen

**Figure 2:**
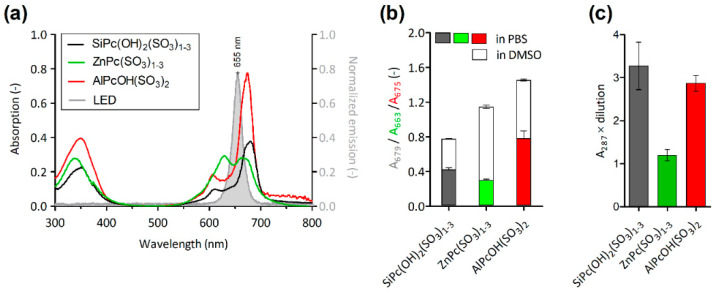
Phthalocyanine absorption spectra, aggregation state and O_2_(^1^∆_g_) production. (**a**) Absorption spectra of Pc derivatives in PBS (10 µg mL^−1^; red, green and black lines) and emission spectrum of LED light (gray peak) normalized to the maximal Pc absorption. (**b**) Absorption maxima of Pc derivatives in PBS and in DMSO (*n* = 3). (**c**) Relative production of O_2_(^1^∆_g_) during photoactivation of Pc derivatives in iodide solution (*n* = 3).

The absorption spectra of Pcs contained maxima at 663 nm for ZnPc(SO_3_)_1-3_, at 675 nm for AlPcOH(SO_3_)_2_ and at 679 nm for SiPc(OH)_2_(SO_3_)_1-3_. The spectrum of ZnPc(SO_3_)_1-3_ contained a second maxima at 630 nm, suggesting the presence of Pc dimers [22], which were not detected in the spectra of the other derivatives (Figure 2a). The absorption peaks overlapped with the emission peak of the LEDs utilized for the photodynamic treatment. The dissolution of Pcs in DMSO led to the increase in their absorbance 1.8× for SiPc(OH)_2_(SO_3_)_1-3_, 1.9× for AlPcOH(SO_3_)_2_ and 3.8× for ZnPc(SO_3_)_1-3_, reflecting the different aggregation statuses of the derivatives in PBS (Figure 2b).

The photoactivation of Pcs leads to the generation of highly reactive O_2_(^1^∆_g_), which is responsible for the PDI of prions [10]. The potency of the studied derivatives to produce O_2_(^1^∆_g_) was compared under the same experimental conditions. The amount of O_2_(^1^∆_g_) was estimated by the spectrophotometric method [23]. SiPc(OH)_2_(SO_3_)_1-3_ and AlPcOH(SO_3_)_2_ produced similar relative (A_287_ × dilution) amounts of O_2_(^1^∆_g_), 3.3 ± 0.5 and 2.9 ± 0.2, respectively. The amount of O_2_(^1^∆_g_) released by ZnPc(SO_3_)_1-3_ (1.2 ± 0.1) was approximately 2.7× and 2.4× lower, respectively (Figure 2c). Neither the illuminated iodide solution alone nor the Pcs in iodide solution left in the dark produced false positive reactions.

### 2.2. Photodynamic Elimination of Prion Epitopes

#### 2.2.1. Demonstration of Epitopes Elimination by a Panel of Prion Antibodies

**Figure 3:**
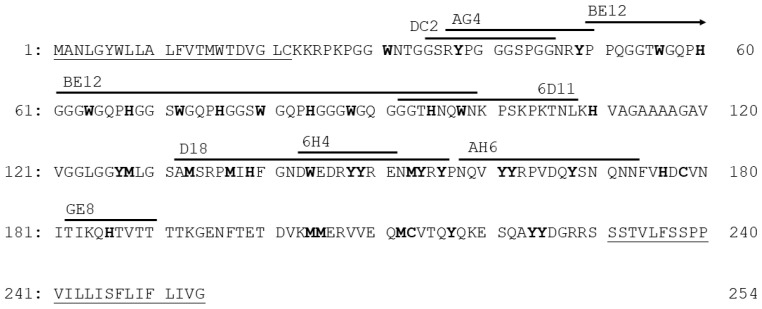
Amino acid sequence of mouse prion protein (UniProt P04925) with the location of epitopes of monoclonal antibodies DC2 (aa35-46), AG4 (aa37-50), BE12 (aa50-99), 6D11 (aa93-109), D18 (aa132-156), 6H4 (aa144-152), AH6 (aa159-174) and GE8 (aa183-191). Underlined letters mark signal sequences which are cleaved off. The amino acid residues prone to oxidation by O_2_(^1^∆_g_) are depicted in bold.

**Figure 4:**
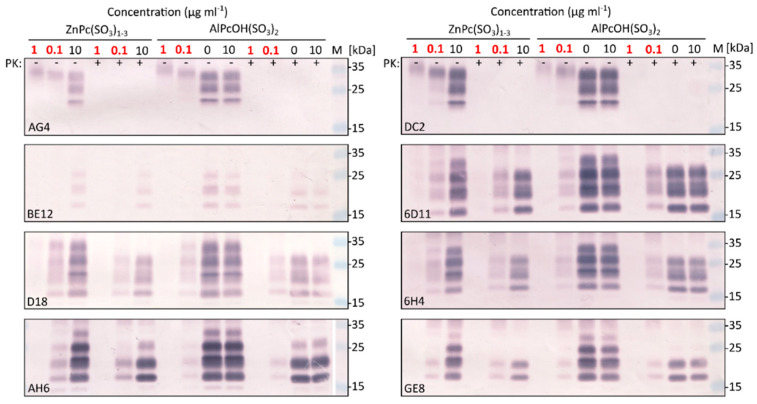
Photodynamic elimination of PrP^C^ and/or PrP^TSE^ epitopes proved by failure of antibodies binding. RML brain homogenate with 0.1 or 1 µg mL^−1^ ZnPc(SO_3_)_1-3_ or AlPcOH(SO_3_)_2_ was illuminated for 90 min (red numbers) and control aliquots kept in dark either non-treated or with 10 µg mL^−1^ of the Pc derivative (black numbers). One aliquot of each sample was cleaved with proteinase K (PK; +) to remove protease sensitive PrP^C^ and visualize PrP^TSE^. PK treatment cleaves off the N-terminal part of PrP^TSE^, resulting in its higher electrophoretic mobility and removal of DC2 and AG4 epitopes. The second aliquot was left intact (-) and contained both PrP^C^ and PrP^TSE^. The samples were analyzed by Western blot with antibodies against different parts of PrP molecule (DC2, AG4, BE12, 6D11, D18, 6H4, AH6, GE8). The photodynamic elimination of the epitopes was documented for all tested antibodies. The figure is a representative of three independent experiments.

The locations of the epitopes of nine prion monoclonal antibodies utilized in the study are depicted in Figure 3. Each epitope contains at least one O_2_(^1^∆_g_) sensitive amino acid residue. The photodynamic treatment of 1% (*w*/*v*) RML brain homogenate with ZnPc(SO_3_)_1-3_ or AlPcOH(SO_3_)_2_ followed a similar pattern for all tested antibodies. The Pcs at the concentration 0.1 µg mL^−1^ diminished and at the concentration 1 µg mL^−1^ fully eliminated the signal of proteinase K resistant PrP^TSE^ of all tested antibodies (Figure 4). At the same time, the binding of DC2 and AG4 to proteinase-K-sensitive diglycosylated isoform of PrP^C^ was not fully abolished, and the protein displayed slightly lower electrophoretic mobility (Figure 4). No change in the signal was recorded in the control aliquots containing the Pcs but kept in the dark.

#### 2.2.2. Differences in the Potency of Phthalocyanine Derivatives and in the Sensitivity of Prion Strains

**Figure 5:**
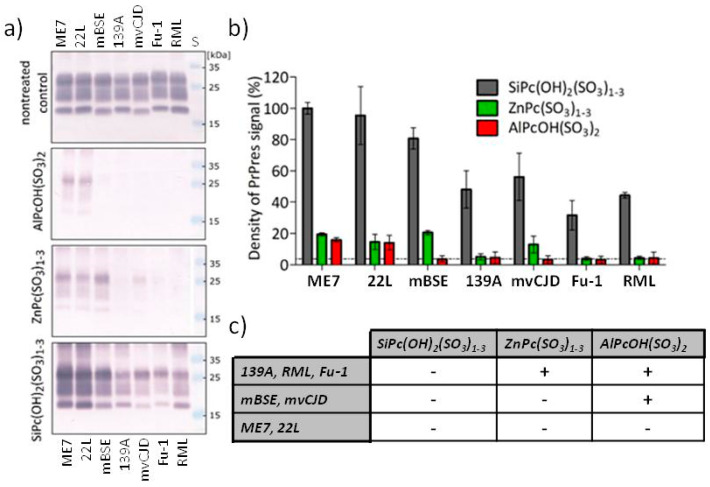
Photodynamic elimination of PrP^TSE^ signal by Pc derivatives. (**a**) Western blot analysis of photodynamic elimination of PrP^TSE^ signal induced by photoactivation with AlPcOH(SO_3_)_2_, ZnPc(SO_3_)_1-3_ and SiPc(OH)_2_(SO_3_)_1-3_. PrP^TSE^ in the brain homogenates infected by different prion strains (ME7, 22L, mBSE, 139A, mvCJD, Fu-1, RML) was detected using the antibody 6D11 as proteinase K-resistant fragments PrPres. S; molecular weight standard. The Western blots are representative of three independent experiments. (**b**) Densitometry evaluation of the PrPres signal on Western blot membranes (*n* = 3). Dotted line represents the background signal. (**c**) Discrimination of the prion strains based on their sensitivity to photodynamic treatment by Pc derivatives. The strain was considered sensitive (+) when the average signal of PrPres on the Western blot reduced by SD is lower than the background.

The potency of Pcs derivatives in the photodynamic PrP^TSE^ epitope elimination was compared at concentration 1 µg mL^−1^. AlPcOH(SO_3_)_2_ was able to decrease the PrPres signal below the detection limit of the Western blot for the mBSE, mvCJD, Fu-1, 139A and RML strains. The less efficient ZnPc(SO_3_)_1-3_ removed the signal for the Fu-1, 139A and RML strains. In contrast, SiPc(OH)_2_(SO_3_)_1-3_ did not eliminated the PrPres signal of any of the tested strains (Figure 5). ME7 and 22L were the least susceptible strains to photodynamic treatment and resisted PrPres signal elimination under the tested conditions. The PrPres signals of the mBSE and mvCJD strains were eliminated only by the most efficient AlPcOH(SO_3_)_2_. The PrPres signals of strains 139A, RML and Fu-1 were removed by both AlPcOH(SO_3_)_2_ and ZnPc(SO_3_)_1-3_. The semi-quantitative estimation of the remaining PrPres Western blot signals was performed by densitometry (Figure 5b). The residual signal after the treatment with AlPcOH(SO_3_)_2_ or ZnPc(SO_3_)_1-3_ was between 0 and 20% for all tested prion strains. The signal after the treatment with SiPc(OH)_2_(SO_3_)_1-3_ varied between 35 and 100%. These results allow discrimination of the prion strains into three groups according to their sensitivity to the PrP^TSE^ epitope elimination (Figure 5c). 

## 3. Discussion

Previously, we have demonstrated that the photodynamic treatment of RML prions by AlPcOH(SO_3_)_2_ leads to a time- and dose-dependent disappearance of PrP^TSE^ signal on Western blots [9,10]. The elimination of the PrP^TSE^ epitopes was accompanied by a significant decrease in prion infectivity for susceptible CAD5 cells. In the follow up study, we have improved the treatment effectiveness by the utilization of an optimized light source. The concentration of AlPcOH(SO_3_)_2_ needed to decrease the PrP^TSE^ Western blot signal by 50% was as low as 22 nM [10]. The treatment significantly reduced the infectivity of RML prions adsorbed onto plastic, suggesting its possible potential in the sanitization of prion-contaminated surfaces. Importantly, the prion infectivity of photodynamically treated RML brain homogenate was diminished by more than 4 log_10_ in the mouse bioassay [10]. At the same time, the infectivity of the sample with AlPcOH(SO_3_)_2_ left in dark did not change confirming that the effect was not caused by the presence of Pc itself. Caughey et al. demonstrated that the direct anti-prion activity of Pc derivatives (not dependent on photoactivity) correlated with their propensity for self-aggregation in aqueous media [16]. However, the self-aggregation of Pcs is known to inhibit their photoactivity as only monomer Pc molecules are capable to generate O_2_(^1^∆_g_) [22]. This raises the question if the AlPcOH(SO_3_)_2_ utilized in our previous studies was efficient in PrP^TSE^ oxidation just due to its low self-aggregation and high O_2_(^1^∆_g_) production. 

To learn more about the importance of Pc characteristics for photodynamic inactivation of prions, we utilized three Pcs differing in sulfonation, coordinated central metal, level of aggregation and O_2_(^1^∆_g_) production. While AlPcOH(SO_3_)_2_ was exactly di-sulfonated, ZnPc(SO_3_)_1-3_ and SiPc(OH)_2_(SO_3_)_1-3_ were mixes of mono-, di- and tri-sulfonated molecules. The absorption spectra of AlPcOH(SO_3_)_2_ and SiPc(OH)_2_(SO_3_)_1-3_ in PBS suggested that the Pcs were predominantly in the monomeric state. In contrast, the spectra of ZnPc(SO_3_)_1-3_ implied a substantial presence of dimers [22]. Likewise, the dissolution of the Pcs in DMSO led to a notably higher increase in the ZnPc(SO_3_)_1-3_ Q band absorbance, confirming its higher aggregation in PBS [22]. The higher aggregation of ZnPc(SO_3_)_1-3_ correlated with its markedly lower photogeneration of O_2_(^1^∆_g_) in comparison to the other two derivatives. The O_2_(^1^∆_g_) production by SiPc(OH)_2_(SO_3_)_1-3_ was slightly higher than by AlPcOH(SO_3_)_2_, as reported earlier [10]. 

All our previous inactivation studies were carried out with the RML strain of mouse-adapted scrapie. However, prion strains can substantially differ in their susceptibility to denaturation and inactivation [6,7,8]. To find out whether this is also the case for the photodynamic oxidation, we studied its effect on seven laboratory strains of prions. All strains were propagated in CD-1 mice and as a source of prions served identically prepared brain homogenates of symptomatic animals. 

In our previous studies, we have used antibodies DC2 and AH6 to demonstrate the disappearance of PrP^TSE^ signal on Western blots [9,10]. To verify that the photodynamic treatment also affects other parts of the PrP^TSE^ molecule, we utilized a panel of antibodies directed against different parts of the protein. The treatment caused similar changes in the binding of all tested antibodies, suggesting that the photodynamic oxidation affects the entire PrP^TSE^ molecule. This finding seems to correlate with relatively even spread of O_2_(^1^∆_g_)-sensitive amino acid residues [21] along the protein sequence. The sensitivity of the strains to the photodynamic oxidation was monitored using the antibody 6D11. The 6D11 epitope is located at the beginning of proteinase K-resistant part of PrP^TSE^, contains two amino acid residues sensitive to O_2_(^1^∆_g_) and gave robust Western blot signal. 

The treatment of the strains with Pc derivatives led to a reproducible pattern. ZnPc(SO_3_)_1-3_ and AlPcOH(SO_3_)_2_ eliminated the PrP^TSE^ signal in three and five tested prion strains, respectively. In contrast, identical treatment with SiPc(OH)_2_(SO_3_)_1-3_ did not eliminate the signal in any out of seven strains tested, despite it having generated the highest level of O_2_(^1^∆_g_). These data suggest that the effectiveness of the photodynamic oxidation of PrP^TSE^ is not entirely dependent on the level of produced O_2_(^1^∆_g_) and that the prion strains differ in their susceptibility to photodynamic oxidation. The half-life of O_2_(^1^∆_g_) is just 3.5 µs [24], and it is able to oxidize targets only in close proximity to the site of its production [25]. Our previous studies with AlPcOH(SO_3_)_2_ demonstrated that the signal of PrP^TSE^ is more readily removed than the signal of PrP^C^ [9,10]. A plausible explanation of this phenomenon may be that the multimeric beta sheet rich structure of PrP^TSE^ provides higher affinity binding sites for AlPcOH(SO_3_)_2_ than PrP^C^ and that the Pc binding leads to a more effective local production of O_2_(^1^∆_g_). The local production of O_2_(^1^∆_g_) was proposed as a cause of the photodynamic suppression of β-amyloid aggregation by porphyrins [26]. Both π–π and electrostatic interactions were involved in the binding of Pcs tetrasulfonate derivatives to recombinant hamster prion protein [18]. It is tempting to speculate that two hydroxyl groups of SiPc(OH)_2_(SO_3_)_1-3_ which are protruding above and below the planar Pc molecule and are known to prevent its self-aggregation may also prevent its π–π interactions with PrP^TSE^ [27]. The lack of local O_2_(^1^∆_g_) production, then, may make SiPc(OH)_2_(SO_3_)_1-3_ the least effective derivative in removing PrP^TSE^ epitopes. In contrast, the binding of less populated ZnPc(SO_3_)_1-3_ monomers to PrP^TSE^ by π–π interactions is preserved, causing its higher efficacy despite significantly lower overall O_2_(^1^∆_g_) production. The highest effectiveness of AlPcOH(SO_3_)_2_ seems to correlate with its ability to participate in π–π interactions and to produce a high level of O_2_(^1^∆_g_). 

The studied prion strains could be divided into three groups differing in their sensitivity to Pc-induced photodynamic oxidation. Most resistant were the mouse-adapted sheep scrapie strains ME7 and 22L. The second group consisted of mBSE and mvCJD. The least resistant strains were RML, 139A and Fu-1. The grouping seemed to follow strain-specific characteristics as the strains known to be related, such as mBSE and mvCJD or RML and 139A, clustered together. The different susceptibility of the strains to the photodynamic oxidation probably stem from diverse conformation of the associated PrP^TSE^. Both the size and stability of PrP^TSE^ aggregates, surface accessibility of sensitive amino acid residues and perhaps also the ability to interact with Pcs may play important roles. For example, RML prions are smaller and less stable than ME7 prions [8], and according to our data, they are also more prone to photodynamic oxidation. 

In conclusion, our data document that the sensitivity of PrP^TSE^ to Pc induced photodynamic oxidation is prion-strain-specific. It underlines the requirement of using a range of relevant prion strains for effectiveness validation of photodynamic prion inactivation procedures. In addition, our finding offers a new way to discriminate prion strains in vitro. The photodynamic treatment is straightforward, and the removal of PrP^TSE^ epitopes can be classified simply as yes or no. An important finding of our study is that the efficacy of Pc derivatives does not depend only on the level of O_2_(^1^∆_g_) production but also on their structural features. Most likely the binding of Pcs to PrP^TSE^, possibly via π–π interactions, is essential for more effective local production of O_2_(^1^∆_g_). This finding warrants further studies of Pc interactions with PrP^TSE^, which may help to improve the effectiveness of the treatment to the level allowing its utilization as a convenient method for prion decontamination. 

## 4. Materials and Methods

### 4.1. Phthalocyanine Derivatives Characterization

The phthalocyanine derivatives AlPcOH(SO_3_)_2,_ ZnPc(SO_3_)_1-3_ and SiPc(OH)_2_(SO_3_)_1-3_ were provided by Centre for Organic Chemistry (Rybitvi, Czech Republic). The Pcs were diluted in PBS pH 7.4 to a concentration 100 µg mL^−1^. Spectra of Pc derivatives were recorded for 10 µg mL^−1^ solution in PBS and in DMSO (*n* = 2) to monitor their level of aggregation [22]. The production of singlet oxygen was measured using the iodide method [23]. Briefly, the Pcs were diluted (1 µg mL^−1^) in the iodide solution (80 mM potassium iodide, 42 mM potassium dihydrogenphosphate, 27 µM ammonium molybdate, pH 6.2) and illuminated in a 96-well plate for 90 min as described in Section 4.3. The generated O_2_(^1^∆_g_) oxidize I^-^ to I_3_^-^ which absorbs at 287 and 351 nm, and its absorption is proportional to O_2_(^1^∆_g_) concentration. Controls were represented by illuminated iodide solution and the solution with Pcs kept in dark. Spectrometric measurements were performed by Biochrom WPA Biowave S2100 Diode Array spectrophotometer in UV cuvettes (Brand GmbH+CO, Liebenscheid, Germany) of 10 mm path length.

### 4.2. Tissue Samples

The archive frozen 10% brain homogenates (*w*/*v* in PBS pH 7.4, 2 mM EDTA, 1 mM PMSF) of symptomatic CD1 mice infected with the strains ME7, 22L, 139A, RML, Fu-1, mvCJD and mBSE were utilized as the source of prions. The mice were inoculated intracerebrally with 1% infectious brain homogenate, as described previously [10]. After reaching the terminal disease phase, the animals were sacrificed, and their tissues were harvested. The experiments were carried out in accordance with the good animal practice recommended by the Federation of Laboratory Animal Science Associations and approved by the Charles University Committee on the Ethics of Animal Experiments.

### 4.3. Photodynamic Oxidation of PrP^TSE^

Photodynamic treatment of the brain homogenates was performed as described previously [10]. Briefly, 1% (*w*/*v*) brain homogenate was mixed (9:1) with Pc solution to obtain its final concentration 1 µg mL^−1^. The illumination was performed in 96-well plate using a diode array light source equipped with LEDs of 100 mW power and the emission maximum at 655 nm (W53SRC/E, Kingbright, Taiwan) for 90 min. One aliquot of the sample was treated with 50 µg mL^−1^ proteinase K for 30 min at 37 °C to prepare proteinase K-resistant fraction of PrP^TSE^.

### 4.4. Western Blotting

Proteins resolved by sodium dodecyl sulphate polyacrylamide gel electrophoresis in 10% polyacrylamide gels were transferred onto 0.2 µm nitrocellulose membrane as described previously [28]. Proteinase K-resistant PrP^TSE^ was probed with mouse monoclonal antibodies (0.5 µg mL^−1^) AG4, BE12, AH6, GE8 (cat. no. RC059, RC060, RC058, RC061, TSE Resource Center, Roslin, UK), DC2 (Blood Transfusion Center of Slovenia, Ljubljana, Republic of Slovenia), 6D11 (cat. no. 808002, BioLegend, San Diego, CA, USA), 6H4 (cat. no. 7500996, Thermo Fisher Scientific, Waltham, MA, USA) or human monoclonal antibody D18 (provided by Dr. Burton, The Scripps Research Institute, San Diego, CA, USA). Bound antibodies were visualized using alkaline phosphatase conjugated donkey F(ab´)_2_ fragment anti-mouse IgG (0.22 µg mL^−1^, cat. no. 715-056-150, Jackson ImunnoResearch, La Jolla, CA, USA) or mouse anti-human IgG (1:1000, cat. no. 9042-04, Southern Biotech, Tuscaloosa, Alabama, USA) and colorimetric detection with BCIP/NBT substrate (Millipore, Burlington, MA, USA). The membranes were digitized on a Canon CanoScan LiDE 220 scanner. The signal of PrP^TSE^ bands (di-, mono- and unglycosylated form) was quantified using the ImageJ software [29]. The statistic and other calculations were performed in GraphPad Prism 5, version 5.03. Values were expressed as mean ± standard deviation (SD). Values with *p* < 0.05 were considered statistically significant.

## Figures and Tables

**Table 1:** Phthalocyanine derivatives utilized in the study.

Phthalocyanine Derivative	Coordinated Metal	Peripheral Sulfonation
SiPc(OH)_2_(SO_3_)_1-3_	HO–Si–OH	R1 ᴧ R2 ᴧ R3 ^1^
ZnPc(SO_3_)_1-3_	Zn	R1 ᴧ R2 ᴧ R3 ^1^
AlPcOH(SO_3_)_2_	Al–OH	R1 ᴧ R2 or R1 ᴧ R3 ^2^

^1^ The mixture of mono-, di- and tri- sulfonated phthalocyanine stereoisomers; ^2^ The mixture of di-sulfonanted phthalocyanine stereoisomers.

## Data Availability

The data supporting the results presented in this study are available on request from the corresponding author.

## Sample Availability

Samples of the compounds (AlPcOH(SO_3_)_2_, ZnPc(SO_3_)_1-3_, SiPc(OH)_2_(SO_3_)_1-3_) are available on request from the corresponding author.
